# Executive summary of the 14th HHT international scientific conference

**DOI:** 10.1007/s10456-023-09886-5

**Published:** 2023-09-11

**Authors:** Roxana Ola, Josefien Hessels, Adrienne Hammill, Cassi Friday, Marianne Clancy, Hanny Al-Samkari, Stryder Meadows, Vivek Iyer, Rosemary Akhurst

**Affiliations:** 1 Cardiovascular Pharmacology Mannheim (EPM), European Center for Angioscience (ECAS), Medical Faculty Mannheim, Heidelberg University, Heidelberg, Germany; 2 Department of Pulmonology, St. Antonius Hospital, Nieuwegein, The Netherlands; 3 Cancer and Blood Diseases Institute, Division of Hematology, Cincinnati Children’s Hospital Medical Center, Cincinnati, OH USA; 4 Department of Pediatrics, University of Cincinnati College of Medicine, Cincinnati, OH USA; 5 HHT Foundation International, Inc (Cure HHT), Monkton, MD USA; 6 Massachusetts General Hospital, Harvard Medical School, Boston, MA USA; 7 Cell and Molecular Biology Department, Tulane Brain Institute, Tulane University, New Orleans, LA USA; 8 Division of Pulmonary and Critical Care Medicine, Mayo Clinic, Rochester, MN USA; 9 Helen Diller Family Comprehensive Cancer Center and Department of Anatomy, University of California, San Francisco (UCSF), San Francisco, CA USA

**Keywords:** HHT—Hereditary Haemorrhagic Telangiectasia, BMP—Bone Morphogenic Protein, ENG—Endoglin, ALK1—Activin receptor-like kinase 1, AVM—Arteriovenous Malformation, Epistaxis

## Abstract

Hereditary Hemorrhagic Telangiectasia (HHT) is an autosomal dominant vascular disorder characterized by small, dilated clustered vessels (telangiectasias) and by larger visceral arteriovenous malformations (AVMs), which directly connect the feeding arteries with the draining veins. These lesions are fragile, prone to rupture, and lead to recurrent epistaxis and/or internal hemorrhage among other complications. Germline heterozygous loss-of-function (LOF) mutations in Bone Morphogenic Protein 9 (BMP9) and BMP10 signaling pathway genes (endoglin-*ENG*, activin like kinase 1 *ACVRL1* aka *ALK1,* and *SMAD4*) cause different subtypes of HHT (HHT1, HHT2 and HHT-juvenile polyposis (JP)) and have a worldwide combined incidence of about 1:5000. Expert clinicians and international scientists gathered in Cascais, Portugal from September 29th to October 2^nd^, 2022 to present the latest scientific research in the HHT field and novel treatment strategies for people living with HHT. During the largest HHT scientific conference yet, participants included 293 in person and 46 virtually. An impressive 209 abstracts were accepted to the meeting and 59 were selected for oral presentations. The remaining 150 abstracts were presented during judged poster sessions. This review article summarizes the basic and clinical abstracts selected as oral presentations with their new observations and discoveries as well as surrounding discussion and debate. Two discussion-based workshops were also held during the conference, each focusing on mechanisms and clinical perspectives in either AVM formation and progression or current and future therapies for HHT. Our hope is that this paper will represent the current progress and the remaining unanswered questions surrounding HHT, in order to serve as an update for those within the field and an invitation to those scientists and clinicians as yet outside of the field of HHT.

## Summary of basic science talks

Roxana Ola

## New models to study HHT

In the last few years, several groups generated murine models for HHT and uncovered that AVMs are exclusively a pathogenic feature of canonical BMP9/10 signaling through Alk1 and Eng receptors, Smad1/5 transcriptional factors and the Smad4 transcriptional effector in endothelial cells (ECs) [1–10]. Extensive characterization of the murine vascular lesions in these mouse models revealed cellular similarities to human HHT lesions: changes in EC fate and size together with an aberrant smooth muscle actin coverage, increased proliferation and defective EC migration against the bloodstream [1–9, 11]. Yet, the exact underlying aberrant cell event and the responsible molecular mechanisms driving AVM formation and the enlargement of lesions remain poorly understood. To advance the pathophysiology of HHT, novel disease models of HHT with the potential of enabling further understanding as well as drug discovery and testing were presented in this year’s meeting.

While the groups of Mummery **(O16)** and Hughes **(O21)** developed preclinical human models of HHT, the group of Paul Oh developed a novel mouse model that allows a spatial–temporal induction of brain AVMs **(O24)**. Using a mosaic depletion of *ALK1* in primary ECs grown in a newly developed HHT-on-a-chip microfluidic model, Fang et al. **(O21)** could model AVM formation and identified that AVMs comprised a mixture of *ALK1*-intact and *ALK1*-deficient ECs. Furthermore, they successfully provided evidence of using this system for drug testing and evaluation of pazopanib treatment resulted in successful prevention of AVM formation in their system.

Interestingly, Orlova et al. (**O16**) generated isogenic diseased and healthy human induced pluripotent stem cells from a rare mosaic HHT1 patient (HHT1^c.1678C>T^-hiPSC-ECs). Culturing these cells in a 3D organ-on-chip device under microfluidic flow resulted in lumenized, leaky and disorganized vessels with decreased pericyte coverage resembling* ENG* haploinsufficiency. This humanized preclinical model might represent a valuable tool for personalized HHT treatment.

Scherschinski et al. **(O24)** successfully created a robust brain AVM mouse model by stereotactically conditionally depleting *Alk1* specifically in the brain. This mouse model showed an improved long-term survival that may serve as a framework to accelerate the ongoing investigations of brain AVM pathogenesis and preclinical testing of novel therapeutics.

## Implication of other cell types in AVM pathogenesis

While in previous HHT conferences researchers focused on the contribution of macrophages and inflammatory cells to AVM pathogenesis, this year, researchers showed new interest in elucidating how dysfunctional endothelium within the AVM impacts the neighboring mural cells.

Loss of canonical BMP9/10 signaling in the neonatal endothelium leads to impaired coverage of pericytes exclusively within the AVMs [6–8, 12]. As AVMs form in regions of high flow [7–9, 11] and physiological flow maintains vascular quiescence by regulation of key factors mediating pericyte recruitment to ECs (Transforming growth factor β (TGF-β), Platelet-derived growth factor B (PDGFB) and Jagged (JAG) [11], all together these findings emphasize that disrupted flow-mediated pericyte coverage is yet another hallmark contributing to AVM pathogenesis.

Interestingly, Lebrin’s group **(O22)** found that loss of *Eng* in adult mice led to impaired attachment of ensheathing (EP) and mesh (MP) pericytes at the pre-capillary arterioles causing impaired vasoconstriction in response to electrical stimuli. Interestingly and surprisingly, loss of EC *Eng* also impaired the neuro-vascular coupling in the barrel cortex (a region of the somatosensory cortex) under whisker stimulation. Therefore, Thalgott et al. proposed that EC endoglin modulates mural cell coverage and functions, but also the vasoreactivity to changes in neural activity by maintaining intact TGF-β signaling in mural cells **(O22)**.

Su H and Ola’s groups emphasized that platelet-derived growth factor ß (Pdgfb) signaling through its receptor Pdgfrb protects the EC against AVMs (**O18**, **O29**). Genetic disruption of Pdgfb-Pdgfrb signaling either at the level of the receptor expressed by the mural cells in adult mice in the presence of angiogenic stimuli (**O18**) or by depleting the Pdgfb ligand in neonatal ECs (**O29**) results in formation of AVM-like structures in the brain and developing organs, respectively. Furthermore, impaired Pdgfrb signaling exacerbated the brain AVM phenotype in adult mice with EC LOF of *Eng* (**O18**). From the mechanistic point of view, Lin et al. identified that SMAD4 signaling in ECs is required for either BMP9 or flow-induced angiocrine Pdgfb stimulation of pericyte recruitment in a paracrine manner (**O29**).

## BMP9 and BMP10 signalling in organotypic vascular homeostasis

Novel developments in the field of angiogenesis emphasize that organ-specific EC quiescence is the critical bottleneck for a healthy lifespan as vascular disorders develop often in time due to loss of quiescent EC phenotype of fully mature, differentiated ECs with tissue specific consequences. Similar to other vascular anomalies, even though the receptors are expressed by all Ecs, loss of function genetic variants in *ALK1* or *ENG* (encountered in 90% of HHT patients) cause localized AVMs in the liver or lungs, respectively[13]. Yet, the molecular basis for these organotypic effects of BMP9/10 signaling are far from being understood.

Work from Bailly’s lab was focused on understanding the role of BMP9 and BMP10 ligands in organotypic vascular homeostasis in liver, lung, and heart. Interestingly, depending on the genetic background, loss of BMP9 alone was sufficient to induce vessel enlargement and spontaneous AVMs in the 129/Ola background **(O23)** but not in C57BL/6 mice, where BMP9 and BMP10 seem to play redundant functions under physiological conditions **(O25)**. However, in the latter model, the combined deficiency of *Bmp9* and *Bmp10* in adult mice led to progressive development of high-output heart failure and lung inflammation. Under pathological conditions, such as chronic hypoxia, Bouvard et al. highlighted differential roles for BMP ligands, with BMP9 contributing to pulmonary vascular remodeling, while BMP10 played a key role in hypoxia-induced cardiac remodeling **(O25)**.

Focusing on liver homeostasis, Desroches-Castan et al., identified that BMP9 is required for maintenance of liver sinusoidal EC (LSEC) differentiation, Notch signaling activation and cell cycle arrest **(O23)**. Work from the same lab **(**Ricard et al., **O26)** provided further evidence that downstream of BMP9, ALK1 is required for liver homeostasis and function, as *Alk1* deficiency in adult ECs leads to loss of sinusoidal EC identity due to loss of EC fenestration with an impact on neighboring stellate cells and hepatocytes.

In an attempt to identify a correlation between endoglin expression levels and organotypic clinical manifestation in HHT1, Lebrin’s group (Thalgott et al., **O28**) measured the *Eng* mRNA and Eng protein levels in different vascular beds along with the response of these organotypic ECs to VEGF and BMP9 induced Akt and Smad1/5 activation, respectively. Interestingly, the results revealed a critical *Eng* threshold compatible with the haploinsufficiency model, below which ECs independent of their origin, exhibited abnormal responses to VEGF, supporting the development of drugs promoting *Eng* expression as potentially protective against AVM development.

## Signaling pathways

Human and mouse genetic data support the concept that impaired canonical BMP9 signaling through Smad4 leads to HHT. Yet, the HHT causative genes *ALK1* and *ENG* together with mutations in *BMPRII* have been also linked to development of pulmonary arterial hypertension (PAH). Whether the cellular and molecular mechanisms are similar or different, and which environmental factors contribute to the disease progression remains to be established.

Bailly’s lab **(O20)** studied the impact of heterozygous loss of *ALK1* on gene regulation by comparing the transcriptome of HHT2 and PAH-derived ECs in basal conditions versus BMP9 and BMP10 stimulation. Interestingly, BMP9 and BMP10 ligands seem to regulate similar transcriptomes, irrespective of *ALK1* mutation. In PAH-derived ECs, but not in HHT2-derived ECs when compared to the healthy donor-derived ECs, Al Tabosh et al. identified strong transcriptional changes. However, the authors attributed these changes to second hits (mutation/inflammation) present in the lung microenvironment.

Akhurst ‘s group **(O27)** found that the protein tyrosine phosphatase non-receptor type 14 (PTPN14), an already described genetic modifier of pulmonary AVMs [14], modulates BMP9 signaling pathway in ECs by directly binding to Smad4 and protecting Smad4 from undergoing ubiquitination.

Circulating BMP9/10 heterodimers, the dominant form of BMP9 and BMP10 in the blood, are required to activate ALK1 signaling on the endothelium surface. Work from Hinck’s lab **(O19)** investigated the structural basis for the signaling activity of BMP9 and BMP9 as monomers to show that the dimer:monomer ratio was lower for proBMP9 compared to proBMP10, moreover BMP10 monomers exerted reduced pSmad1 inactivation in comparison with BMP10 dimers. The propensity of BMP9 and BMP10 to form monomers, and the reduced activity of the BMP10 monomer, may be relevant to the formation of the BMP9/10 heterodimer.

## Summary of clinical talks

Josefien Hessels and Adrienne Hammill

## HHT diagnosis

HHT is diagnosed clinically using the Curaçao criteria including: spontaneous recurrent epistaxis, telangiectases at characteristic sites, visceral vascular lesions, and first-degree relative(s) with HHT [15]. HHT symptoms develop over time and may not (yet) be present in children with HHT [16, 17]. Bartra and colleagues (**O36**) retrospectively reviewed 184 patients with suspected or confirmed HHT, assessing the presence of telangiectases and the rate of increase with age using linear regression. A rate of increasing telangiectasia count of 1.4/year starting at age 21.2 was found. Pollak and colleagues (**O41**) assessed the accuracy of the clinical Curaçao criteria in 165 children with genetically confirmed HHT. A first-degree relative with HHT was present in 95%, epistaxis in 58%, typical telangiectasia in 32%, and visceral vascular malformation in 38%. Altogether, 25% of the children met 3–4 criteria, 40% two criteria and 35% less than two criteria. This reminds us that the Curaçao criteria cannot sufficiently exclude a diagnosis of HHT in children and rescreening or genetic testing must be considered.

Yusuf et al. (**O46**) analyzed their single center’s data for the effects of race on HHT symptomatology, including epistaxis severity and incidence of AVMs. A retrospective review showed no association of epistaxis severity score with race. However, pulmonary AVM risk was increased 2.3-fold in people of Asian descent as compared to Caucasians (p = 0.03), and brain AVM risk was increased in Hispanic/Latinx 4.8-fold as compared to non-Hispanic/LatinX Caucasians (p < 0.01). Interestingly, these differences persisted even after correction for genotype. It remains unclear if this discrepancy is an issue of access to care, or other environmental factors, and/or to still-unidentified modifier genes.

### Genetic testing in HHT

DeMille et al. (**O43**) presented their ongoing work to develop standardized variant interpretation for HHT. A ClinGen HHT Variant Curation Expert Panel (VCEP) was formed in 2019 and has completed the process of adapting the ACMG-AMP consensus guidelines and proposed a modified variant interpretation and classification process specific to HHT. This will eventually provide a centralized curated database resource for clinicians and researchers to interpret the significance of variants that might be associated with HHT.

It has previously been reported that telangiectasias develop as the result of a second hit in the unaffected allele of the causative mutant gene [18]. Bayrak-Toydemir and colleagues (**O42**) evaluated nasal and dermal telangiectasias for evidence of genetic second hits using a 736 gene next-generation sequencing panel. Five of 9 cases revealed low level (1–2%) somatic mutations in the wild type allele of *ENG* or *ALK1* in HHT1 and HHT2, respectively, including 4 of 15 nasal telangiectasias and 1 of 4 dermal telangiectasias. The sequencing data also identified somatic mutations in other genes in several of the telangiectasias. This group is in the process of testing archival AVM tissues from various organs, including brain, lung, and liver for similar evidence of second hit mutations.

## Bleeding, iron deficiency, and thrombosis in HHT

### Bleeding

Bleeding is known to be a common complication in HHT, from nasal telangiectasia-related epistaxis, to bleeding from gastrointestinal (GI) telangiectasias or AVMs, or even from cutaneous telangiectases. Pericacho and colleagues (**O10**) used two murine models of HHT (both heterozygous mutations in* Eng*^±^ or *Alk*^±^) to evaluate hemostasis. Both models showed abnormalities in endothelial-dependent hemostasis. In *Eng* deficiency, platelet-endothelial adhesion was impaired, with a resultant reduction in thrombus stability; while in *Alk1* deficiency, alterations in fibrinolysis were noted with decreased plasma PAI-1 and increased t-PA, resulting in accelerated thrombus breakdown. These differential mechanisms could suggest genotype-dependent treatment of bleeding, with potential increased effectiveness of antifibrinolytics for HHT2.

Joyce and colleagues (**O44**) performed whole genome sequencing on 104 participants with HHT in order to search for variants in 75 genes of interest associated with bleeding or hemolytic disorders [19]. 56 gene variants were identified within the cohort, however all were rare with allele frequencies < 0.003. Those patients with higher bleeding severity, previously attributed to HHT alone, were found to have more deleterious variants in platelet and coagulation genes, suggesting that more severe HHT bleeding phenotypes may have additional genetic predisposition, and might benefit from further hematologic workup.

Kasthuri et al. (**O11**) presented new evidence regarding an increased prevalence of heavy menstrual bleeding (HMB) in women with HHT. Data was collected via a survey distributed by Cure HHT to assess menstrual bleeding and quality of life. Among the 633 responses, HMB was reported by 74% of women with HHT as compared to 53% in the general female population; among women with HHT of childbearing age, 49% had sought care for HMB and 56% felt that it had a negative impact on their quality of life. Anemia in the past year was reported by 67%, with 79% of these receiving oral iron, 27% IV iron infusion, and 10% blood transfusion.

While it is widely accepted that HHT-related bleeding can lead to development of iron-deficiency anemia, it is possible that other mechanisms are at work. Sharma et al. (**O45**) compared iron deficiency parameters amongst the HHT genotypes. While hemoglobin was not statistically different, higher serum ferritin was found in *ALK1* as compared to *ENG* (median 31 vs 25, p = 0.006) or to *SMAD4* (median 26, p = 0.03) patients. Mean corpuscular volume was lower in *SMAD4* than in *ALK1* median 75 vs 90, p < 0.0001) or *ENG* (median 89, p < 0.0001). Red blood cell counts were higher in *SMAD4* than in *ALK1* or *ENG*, presumably to compensate for the smaller cell size. The authors speculate that these differences are due to SMAD4 role as a hepcidin regulator [20], which might dictate differential responses to iron replacement therapy based on molecular genotype.

### Thrombotic risk in HHT

Kasthuri et al. (**O14**) assessed thrombotic risk in HHT and its relation to iron-deficiency anemia (IDA), by comparing patients with HHT and IDA to those with HHT only, IDA only, and healthy controls. Patients with HHT and IDA had a statistically significant increase in D-dimer as compared to control subjects (516.52 vs 210.19, p = 0.049). VEGFA levels were increased in both HHT groups; E-selectin levels appeared higher in the HHT groups but did not reach statistical significance. An angiogenesis protein array assay (performed on 2 patients from each of the groups) revealed a fivefold increase in tissue factor in the HHT groups, with several other proteins showing ≥ 50% differential expression. These findings suggest a procoagulant state in HHT, and the differentially expressed proteins may represent potential targets for therapies in the future.

### Antithrombotic therapies in HHT

Al-Samkari and colleagues (**O9**) performed an extensive scoping review of the literature to assess use of anticoagulation and antiplatelet therapy in patients with HHT. Complications included worsening HHT-related bleeding in 41% of patients, with early discontinuation of antithrombotic therapy in 40% of these (23% overall). Therapies to control bleeding – local ablative therapy or systemic therapies – were attempted in 8.6% of cases.

Next, Al-Samkari and colleagues (**O15**) from 5 centers prospectively observed outcomes of anticoagulation and antiplatelet therapies in patients with HHT. Together they followed 119 patients through 187 antithrombotic therapy episodes: 59 patients (48%) dose-reduced or discontinued therapy prematurely due to worsening bleeding; similar rates of reduction or discontinuation were seen regardless of antithrombotic medication (44–48%), with only single-agent antiplatelet therapy showing a slightly lower likelihood of bleeding (37%). A history of prior GI bleeding was predictive of discontinuation (3.25-fold odds, p = 0.001). Across the board, hemoglobin levels were lower, IV iron infusions and pRBC transfusions increased, and ED visits and admissions increased in the 3 months after starting antithrombotic therapies as opposed to the 3 months prior, confirming that such therapy remains challenging in patients with HHT.

Whitehead et al. (**O13**) presented safety and efficacy of left atrial appendage occlusion (LAAO) for stroke protection in the setting of atrial fibrillation (AF) in patients with HHT, who might not tolerate long-term anticoagulation. In their cohort of 329 consecutive adult patients with a definite diagnosis of HHT, 31 had atrial fibrillation. Eleven of these patients underwent LAAO; 20 control patients did not. Three in each group used anticoagulation. There were 7 ischemic strokes related to AF, with 3 in control patients and 4 in the LAAO group prior to procedure; no strokes were observed in the treatment group post-procedure, though 2.96 would have been expected based on prior frequency. The approach may be particularly useful for those patients who have significantly increased bleeding with anticoagulation therapy.

## Pulmonary arteriovenous malformations

### Screening

Pulmonary arteriovenous malformations (PAVM) are direct connections between the pulmonary artery and pulmonary vein and can develop in different types: simple (one feeding artery), complex (multiple feeding arteries) and diffuse. Lai and colleagues (**O30**) proposed a fourth subtype of PAVM, the telangiectatic PAVM, visible as a ground-glass lesion on chest CT—encountered in 36% of patients. In 10 patients, no vascular PAVM was visible aside from the ground-glass lesions – while having a mild to moderate pulmonary right-to-left shunt (RLS), suggesting that the ground-glass lesion is likely to be a real (telangiectatic) PAVM.

Screening for PAVMs is recommended in individuals with (putative) HHT. In adults, transthoracic contrast echocardiography (TTCE) is the recommended first-line screening method for the presence of PAVMs, including a rescreening interval of 5 years [21]. Previous research indicated that the rescreening interval might be extended safely in patients with initial negative screening [22–26]. Hessels and colleagues (**O33**) presented that in patients without a pulmonary RLS at baseline, no treatable PAVMs were found after five and ten years [27]. Therefore, it might be safe to extend the rescreening interval of patients without a pulmonary RLS to ten years. Blivet and colleagues (**O37**) retrospectively evaluated the number and size of PAVMs on chest CT before and after pregnancy in 30 women. An increase of small PAVMs in 6/30 patients and reperfusion was found in 1/30 patients, further emphasizing the need to repeat follow-up after pregnancy – as recommended in the international guidelines [21].

Fish et al. (**O12**) used their Center’s database of 2310 patients to estimate incidence and prevalence of spontaneous pulmonary AVM rupture in patients with HHT. Over a 25-year period, 801 patients (759 definite, 42 possible) were found to have PAVMs. Spontaneous rupture occurred in 22 patients over an average follow up period of 16.3 years (range 0–25), with calculated prevalence of 2.7% and an annual risk of 0.16% for those with PAVM. Rupture occurred during pregnancy in 5 of the 22 (22.7%); none of the patients had been treated previously and PAVM rupture was the first presentation of HHT in 9 of the patients (40.9%). Eighteen were treated with embolization and 4 with lobectomy. These results again reinforce current screening and treatment guidelines to prevent this feared complication.

In addition to pulmonary symptoms such as hypoxia or hemoptysis, pulmonary AVMs present risk of neurologic complications. Lau and colleagues (**O58**) identified 80 patients (out of 218) with PAVMs, of which 10 displayed stroke episodes. Transient ischemic attack was twice as common in those with PAVM as compared to those without (11.3% vs 5.1%), with a risk of 2.37 times higher after controlling for confounders. In addition, multiple patients reported migraine (21), seizure (5), or brain abscess (3). Future studies are planned to correlate grade of PAVM shunting with neurologic complications to better assess risks.

In order to minimize radiation exposure when possible, in a population exposed to a significant radiation dose [28], Si-Mohamed and colleagues (**O31**) compared ultra-low dose (ULD) chest CT to the reference low dose in 45 HHT patients. PAVMs with a feeding artery diameter of > 2.5 mm were detected with a sensitivity of 100% and a specificity of 87%, while the radiation dose was reduced by 91% [29].

### Treatment

Treatment of PAVMs usually consists of endovascular embolization; however, with persistent perfusions and recanalization, the development of additional (systemic) collaterals may occur. Currently, chest CT is the gold standard for follow-up after PAVM embolization. DePietro [30] previously showed that graded TTCE can similarly detect treatable PAVMs in patients after embolization. Curnes and colleagues (**O34**) presented results of the use of TTCE in the early post-treatment period comparing chest CT and graded TTCE at six-month follow-up. Similar results were observed: no treatable PAVMs were found in patients with no or minimal RLS. Latif and colleagues (**O53**) compared the most commonly used agents: coils, Micro Vascular Plug System (MVP) and Amplatzer Vascular Plug (AVP). A lower risk of persistence was found using MVPs [31]. For the treatment of recurrent PAVMs, Si-Mohamed and colleagues (**O57**) evaluated the safety and efficacy of embolization with ethylene vinyl alcohol copolymer (Onyx®) for recurrent PAVMs. A short-term occlusion rate of 100% and a long-term occlusion rate of 60% were found [32]. Onyx® may be considered as a possible treatment option in pre-treated PAVMs. Cadot and colleagues (**O35**) retrospectively evaluated the frequency of systemic collateral supply after PAVM embolization in children and found a high proportion of patients (44%) with collateral supply involvement after a mean follow-up period of 8.2 years. Trerotola and colleagues (**O56**) presented the outcomes of 9 patients with diffuse PAVMs undergoing surgery, including 7 patients with HHT. In four patients, PAVMs were embolized before surgery. Partial lobectomy (n = 3), complete lobectomy (n = 5) or pneumonectomy with expander (n = 1) were performed, with improved oxygen saturation, resolution of symptoms and no major complications at mean follow-up of 5 years. Surgery can be considered in carefully selected cases.

## Hepatic vascular malformations

Screening for hepatic vascular malformations (HVMs) is recommended in adults with definite or suspected HHT [33]. The imaging test of choice is Doppler ultrasonography, but clinical practice also depends on local availability and expertise. In most HHT centers, HHT patients undergo TTCE (transthoracic contrast echocardiogram) as first-line screening for the presence of PAVMs. Singh and colleagues (**O39**) found a predictive association between the presence of right atrial dilation on TTE (transthoracic echocardiogram) and HVMs, which may possibly contribute to selecting patients undergoing additional imaging. The use of phase-contrast MRI (Magnetic Resonance Imaging) as a method to quantify hepatic blood flow in the abdominal aorta and hepatic artery, thereby indicating the degree of vascular shunting, was presented by Mena and colleagues (**O32**). Increased flow volumes were found in patients with HVMs, which were normalized upon anti-angiogenic treatment.

## Brain vascular malformations

International HHT guidelines recommend brain screening in children and adults with HHT, but the need for rescreening remains undefined [33]. Beslow and colleagues (O40) investigated the presence of de novo brain vascular malformations (BVMs) in patients included in the Brain Vascular Malformation Consortium (BVMC) HHT project. In two patients, a capillary malformation was visualized, after an initial negative MRI. One patient, with two BVMs at age 24 years, developed de novo BVM detected at 37 years. Also, within the BVMC HHT project, Kilian and colleagues (**O52**) compared demographic and clinical data of children with BVMs with a cohort of adults with BVMs – and found a higher proportion of pediatric HHT patients symptomatic at presentation, with a higher prevalence of intracranial hemorrhage and seizures on presentation. Surgical resection was the most commonly used treatment method in both children and adults, and post-treatment hemorrhage was uncommon.

The use of endoluminal biopsy of the vessel lumen of BVMs for the characterization of gene expression and blood-flow mediated transcriptional changes was presented by Winkler and colleagues (**O47**), showing a similar gene detection and genome-wide expression compared to tissues acquired from open surgery [34]. This group (**O17**) then used single-cell mRNA sequencing to create a human cerebrovascular cell atlas from adult human brain and AVMs. AVMs were noted to have loss of normal arterio-venous zonation, with emergence of a distinct cell state in the nidus with heightened angiogenic potential. These techniques may provide opportunities for profiling brain AVMs, contributing to precision medicine opportunities in the future.

## Targeted therapies for HHT

### Pre-clinical models identify promising potential therapeutics

Thalgott et al. (**O7**) reported use of several AKT inhibitors in a preclinical mouse model of HHT1 (*Eng*^flox/flox^;*Cdh5*-*Cre*^ERT2^). Perifosine, uprosertib, and VAD044 were administered to.

Tamoxifen induced mice. VAD044 showed the best efficacy in inhibiting retinal AVM formation and by decreasing levels of phospho-S6, a readout of AKT activity. This compound is now in early Phase clinical trials for patients with HHT.

Su et al. (**O8**) presented their work using adeno-associated viral vectors to deliver long transgenes to minimize brain AVM severity in *Eng-*deficient mice. They evaluated 3 different engineered capsids with a dual goal: of increasing the efficacy of delivery into the brain ECs and limiting hepatic toxicity; AAV capsids were delivered either intravenously or intranasally. AAV.cc84 was identified as the best candidate for future human trials, transducing brain ECs with a high efficiency, while minimally infecting hepatocytes via IV route, and transducing brain perivascular cells and nasal epithelium via intranasal delivery without affecting the hepatocytes.

### Clinical trials in HHT

Dupuis-Girod and colleagues (**O2**) presented results of a placebo-controlled phase 3 study in 24 patients across 16 HHT centers in France evaluating safety and efficacy of bevacizumab for severe bleeding in HHT. Per inclusion criteria, all participants required ≥ 4 units of packed red blood cells (pRBC) in the 3 months prior to study enrollment. The primary outcome was change in pRBC requirement after bevacizumab 5 mg/kg given every 2 weeks × 6 doses; patients on placebo showed minimal change (7.6 units to 7.42 units, or 4% decrease) while those on bevacizumab showed a larger difference (12.8 units to 7.1 units, a 44% decrease) but did not meet the study goal of ≥ 50% reduction in transfusion requirement. Concerns were raised about the differences between the placebo and treatment groups at baseline, as well as lack of information about IV iron support throughout the trial.

Kasthuri et al. (**O3**) reported a retrospective study of long-term safety of bevacizumab in 40 patients with HHT. These patients had a median treatment time of 39.5 months and 26 infusions, with most started for epistaxis. While many experienced some adverse events (AEs) during induction, most were infusion-related and transient; these AEs included 5 patients with persistent/worsening hypertension (4 within weeks of first infusion), one thromboembolic event related to a mediport (with symptoms predating infusions), and no persistent proteinuria. Some patients showed mild liver enzyme elevations not requiring change in therapy. Also, some patients after 2–3 years reported unexplained mild cytopenia (leukopenia and thrombocytopenia).

Hessels and colleagues (**O4**) shared results of an open-label pilot study of tacrolimus for treatment of bleeding in HHT [35]. 25 patients with severe bleeding were enrolled, with 5 stopping early (4 due to serious AEs). In the remaining 20, hemoglobin increase was statistically significant (6.1 to 6.7 mmol/L or 9.8 to 10.8 g/dL, p = 0.003) and blood transfusions decreased from a mean of 5 to 1.9; 13 of the 20 patients experienced at least one AE. 95% of patients were satisfied or very satisfied with their experience, and 65% chose to resume drug after the trial was complete.

Faughnan et al. (**O5**) reported results of a placebo-controlled crossover trial of doxycycline for control of epistaxis in HHT [36]. The study was stopped early due to COVID-19, so only 13 patients were randomized to treatment or placebo first. This study did not show any statistically significant differences in the primary outcome of weekly epistaxis duration, although 80% of participants reported “benefit from the trial” and 2 chose to restart doxycycline after the trial was complete. A secondary qualitative analysis revealed 5 patients who responded to treatment, and baseline angiopoietin-2 levels were found to be significantly different between responders and non-responders. This may allow for identification of those patients with HHT who could most benefit from this therapy in the future.

Ahluwalia and colleagues (**O6**) performed a retrospective analysis of patient characteristics for those treated with pazopanib for refractory HHT-related bleeding. Nine of 14 had been treated with bevacizumab previously (mean 35.33 months, range 7–72 months) and 4 of these had been treated with additional anti-angiogenics (all 4 tried doxycycline and 2 also tried pomalidomide). Patients were treated with 200 mg daily initially; 5 patients decreased to 100 mg daily due to side effects.

## Research networks

Shovlin and colleagues (**O48**) presented the timeline and output of the VASCERN-HHT working group; a collaboration between HHT experts in Europe founded in 2016 aimed to guide management in general and specialist care, with output directed to healthcare professionals, HHT patients and medical specialists [37]. Friday and colleagues (**O1**) presented their work with the Chan Zuckerberg Initiative’s Rare as One project to establish a research network for HHT, guided by patient needs, to prioritize projects based on feasibility, impact, and importance. To date this work has produced 8 different workstreams resulting in a list of 31 recommendations to address patient needs over the next 3–5 years. As more potential treatments for HHT become available, research networks for coordinating prospective clinical treatment trials become even more important to provide access to larger numbers of patients and adequately recruit in the rare disease.

## Other topics of interest

### Non-pharmacologic treatment of GI bleeding

Goldman and colleagues (**O54**) presented the results on the use of double-balloon enteroscopy (DBE) with argon plasma coagulation to treat small bowel GI bleeding, and found no significant decrease in hemoglobin or change in transfusion requirements after 12 months. They concluded that DBE-directed treatment of small bowel telangiectases is safe and does not worsen bleeding. The added value of the treatment needs to be further investigated.

### Quality of life

Maiorano and colleagues (**O51**) performed a cross-sectional study about epidemiological and clinical factors contributing to epistaxis severity and QoL. An association was found with hypertension, septal perforation, nocturnal epistaxis, surgery, blood transfusion, and hormonal therapy [38]. Le and colleagues (**O49**) developed and validated a specific measurement tool: QoL-HHT including a 24-item scale [39].

### Grading systems

Soudry and colleagues (**O38**) proposed a novel simplified nasal endoscopy grading system including three grades: mild (with a few punctate lesions), moderate (with multiple telangiectases/ large arteriovenous malformations involving the anterior nasal septum) and severe (with diffuse involvement of the nasal mucosa with telangiectases). However, the audience pointed out that good visualization of the nasal mucosa is difficult in clinical practice and uniform interpretation of lesions may be difficult. McRae and colleagues (**O55**) presented a staging system also including extranasal telangiectases. The Utah telangiectasia assessment for HHT (U.T.A.H.) includes both qualitative and semiquantitative staging for site, severity, and size of telangiectases.

### HHT-like syndromes

A few cases of a rare form of *GDF2*-related HHT have been described [40–42]. Farhan and colleagues (**O50**) reported four patients with *GDF2-*related HHT. In two patients a heterozygous missense variant accompanied with features similar to HHT 1, and in two other patients with larger interstitial deletions with phenotypic mucocutaneous capillary dysplasia and extravascular findings related to the deletion [43].

Guilhem et al. (**O59**) presented a series of HHT-like hepatic vascular abnormalities in patients with capillary malformations (CM)-AVM vascular syndrome. From a database of 43 patients (18 with *RASA1* and 25 with *EPHB4* mutations), 7 out of 20 patients undergoing adequate liver imaging showed abnormal liver findings*.* Interestingly, all of these patients had *EPHB4* variants (6 pathogenic and 1 likely pathogenic). Abnormal liver imaging findings included dilated hepatic artery in all patients. In addition, imaging identified hepatic telangiectases (in 6 cases), arteriovenous shunts (5), portovenous shunts (4), and arterioportal shunts (3). Six of the liver presentations were considered classic for HHT. Therefore, genetic testing, including *EPHB4* is recommended in atypical HHT cases.

## Workshops

Cassi Friday

## Workshop A: AVM formation and progression: mechanistic and clinical perspectives

### Moderated by Drs Hans-Jurgen Mager, Kevin Whitehead, Paul Oh, and Douglas Marchuk

This workshop hosted approximately half the conference attendees and engaged both clinical and basic scientists in exciting discussions with regards to cellular and molecular mechanisms triggering AVM formation, enlargements, and also maintenance. The moderators initiated the workshop by posing five debatable questions to the audience that prompted a thrilling brainstorming session with stimulation of new collaborations and novel ideas worth investigating in the near future.

First, the group discussed implications of Smad4-dependent or -independent factors to HHT. Next, the moderators challenged the audience with the responsible mechanisms for AVM initiation versus AVM maintenance in various tissues, followed by debating AVM organotypicity.

Further on, the conversation turned towards the therapeutic perspectives for HHT. It was debated whether medical therapy should be offered to patients with HHT with extensive microvascular pulmonary AVMs and if liver transplantation should be offered to high output heart failure patients who display a good response to bevacizumab.

It is well known that AVMs tend to develop with an increased prevalence in some organs over others (for instance, lungs over spleen) and there is even an anecdotal observation of regional propensity of AVM formation within organs that needs yet to be clarified (why do AVMs cluster within one specific lung lobe versus the other?). Some AVMs are congenital while others seem to arise over time. Much of the discussion centered around exposure of prevalent organs to environmental factors. For example, the mucosa in the nose/mouth and gastrointestinal tract, or the lungs, are regularly exposed to potential environmental mutagens that may increase AVM progression and persistence compared to other presumably more protected organs.

## Workshop B: current and future therapies for HHT: mechanistic and clinical perspectives

### Moderated by Drs Marcelo Serra, Sophie Dupuis-Girod, Luisa Maria Botella Cubells, and Roxana Ola

The other half of the conference attendees participated in Workshop B which was designed to debate the needs of the HHT community from the perspectives of basic scientists, clinicians, and patients. All three perspectives are equally important for development of new treatments and overall comprehension of HHT mechanisms and outcomes. From the candid discussions, three overarching topics arose: HHT models, gene therapy, and clinical trials.

Several cellular and animal models of HHT have been developed, however none recapitulates perfectly the human HHT manifestations. There is a common agreement within the HHT community that other models are still worth pursuing. Nevertheless, the existent HHT models ‘hands on’ are still of great value in advancing our basic knowledge in disease manifestations and identifying novel potential therapeutic advances.

One suggestion was to publish a community-wide review article describing all known and commonly used HHT models as suitable experimental models as a guide for our internal community and more importantly for reviewers assessing HHT-related grant applications. This would provide confidence in vetted-model systems while at the same time providing a suitable guide to reviewers.

As the field works to intervene within the signaling pathway to ameliorate AVM formation and progression, gene therapy is on the horizon and should also be a focus for researchers. As such, it has been proposed to generate a gene therapy task force in order to advance technology and application for gene therapy in HHT.

Finally, clinical trials were discussed at length for the HHT community. The HHT field is well-poised for clinical trials research and there are currently multiple clinical trials offered (investigational uses of pomalidomide, pazopanib, and VAD044, for example). However, there is a strong desire to leverage our tight-knit, international community to create more robust clinical trials, international collaborations, links to biotech companies, and utilize platform trial designs. Strong discussions took place surrounding the creation of common endpoints and resources across clinical research for HHT in order to better foster international collaborations, meet regulatory compliance in multiple countries, and to strengthen the power and diversity of clinical research for people living with HHT.
